# Experiences of access to general practice in England: qualitative study and implications for the NHS 10 year plan

**DOI:** 10.1136/bmj-2025-087367

**Published:** 2026-01-14

**Authors:** Carol Sinnott, Akbar Ansari, Evleen Price, Katy Horder, Ahmed Alboksmaty, Janet Willars, Jake Beech, Hugh Alderwick, Mary Dixon-Woods

**Affiliations:** 1 THIS Institute, Department of Public Health and Primary Care, University of Cambridge, Cambridge, CB2 0SR, UK; 2 School of Sociological Studies, Politics, and International Relations, University of Sheffield, Sheffield, UK; 3 Nuffield Department of Primary Care Health Sciences, University of Oxford, Oxford, UK; 4 Imperial College London, Institute of Global Health Innovation, London, UK; 5 University of Leicester, Leicester, UK; 6 Global Counsel, London, UK; 7 The Health Foundation, London, UK

## Abstract

**Objective:**

To report experiences and views of patients, carers, and staff on access to general practice in England in the context of major government plans to reform NHS services.

**Design:**

Qualitative interview study.

**Setting:**

Patients and carers in Devon, Medway, Blackpool, Luton, and Lancashire, and NHS general practices in the east of England.

**Participants:**

70 interviews with 41 patients and carers and 29 general practice staff, including general practitioners (GPs), nurses and allied health professionals, practice managers, and administrators. Analysis was based on the constant comparative method, with themes mapped to the three shifts—to digital, to community, and to prevention—proposed in the 10 year plan for England.

**Results:**

Patient participants represented 12 ethnic groups and diverse personal and medical characteristics. The three shifts offered some benefits to participants but also introduced new risks and disadvantages. The shift to greater digitisation in general practice (mainly in the form of online appointment booking systems and access to medical information) offered more convenience for some patients and improved efficiencies. The shift did little to resolve the fundamental scarcity of appointments with a GP, however, and it introduced new forms of disadvantage and exclusion while failing to address what patients were often seeking: human connection and empathy with a GP they knew. The shift from hospital to community based services, with GPs working over greater geographical scale in new neighbourhood based models, was perceived by participants to offer greater capacity for appointments but faced constraints including practical challenges to coordination and organisation. New services encompassing larger areas risked patients feeling unrecognised and unknown at their practice and undermining the long term relationships with GPs that patients valued. Prevention efforts, while accepted as important, were seen as challenged by their tendency to fragment care, oversimplified models focused on single diseases, and consuming capacity that could otherwise be used for contacts initiated by patients. Concern about increased workload for staff at general practices was consistently expressed.

**Conclusions:**

Although improving access to general practice is a stated priority in government plans to reform NHS services, the three proposed shifts may not be what patients are seeking or what practices want in order to support their work. The proposals will require careful design, implementation, and evaluation in collaboration with key stakeholders, to ensure they do not undermine continuity of care nor fragment existing services.

## Introduction

High quality primary care is strongly associated with better functioning of healthcare systems,^1^ ^2^ enabling the needs of most patients to be met by offering first contact care for undifferentiated illness, comprehensive care across the lifespan, gatekeeping of more specialised services, and coordination of care.^1^ ^3^ Although the NHS in England is built on the principle of universal access to general practice,^4^ patients’ experience of access to and continuity of care has worsened in recent years,^5^ ^6^ ^7^ to the extent that a top public priority is making it easier to get appointments at general practices.^8^ Many attempts to improve access over several decades^9^ suggest that few easy solutions to improving access are available, so scrutiny of policy initiatives is imperative.

The UK government’s landmark 10 year plan for health, published in July 2025^10^ is an important example of policy directed at reforming the NHS in England. It features a number of familiar pledges to improve access to general practice services (including same day appointments “for those who need it”), but it also seeks more fundamentally to reimagine how care is delivered in the NHS by proposing three major shifts (table 2, table 3, table 4). All have substantial implications for general practice in England; the first shift (from analogue to digital systems) aims to imitate the self-service models seen in other industries, such as banking and travel. It includes an expanded NHS app positioned as the so-called digital front door of the NHS, with patients able to book and manage appointments, self-refer to some specialist services, and access a range of information about their health to support self-care. The second shift—from hospital to community—seeks a neighbourhood based approach to delivering services,^11^ with general practitioners (GPs) asked to work at greater geographical scale in collaboration with other health and social care staff in multidisciplinary teams, including in new neighbourhood health centres intended to operate as one stop shops. Primary care is also planned to have a central role in the third shift, to prevention, which will seek to use technology, data, genomics, and community resources in more personalised efforts to prevent ill health. All three shifts will mean major changes in the way general practice in England works, the services on offer, and how people access them (table 2, table 3, table 4). These changes include alternatives to traditional appointments with a known GP—for example, appointments with other clinical professionals, providing information to support self-care, and enabling access to care over wider geographies through neighbourhood based models.

**Table 2:** Policy shifts, study findings, and illustrative quotations: analogue to digital

Key elements of policy shift	Study findings	Illustrative quotations
End the 8 am scramble for appointments. Bring back the family doctor Provide same day GP appointments, digitally or by telephone, to people who need them Increase use of digital telephony Add new functionality in the NHS app: My NHS GP: AI-powered advice for non-urgent care and navigation My Specialist: Ability to book tests directly My Consult: Direct booking of certain services My Medicines: Help managing medicines My Vaccines: Vaccination booking and records My Care: Support for long term conditions My Health: See and upload health data My Companion: Support patients’ articulation of health needs My Children: Child health records and advice My Carer: Access to appointment booking and other services on someone else’s behalf Introduce single sign on systems Support practices to adopt ambient voice technology. Introduce a new single patient record (including new AI summarisation tools)	The shift to digital booking systems was generally well advanced across practices, although telephone based systems remained in place in many settings Where digital transformation was most advanced, practices restricted methods for booking appointments other than online Some aspects of digitisation were valued, but what both patients and professionals were often seeking human connection and continuity The shift to digital created new labour for patients, and had potential to exclude some people, create risks, and deepen inequities Shortage of appointments (especially with a GP) was a persistent problem, regardless of mode of access Patients and clinicians both reported challenges of the dehumanised character of digital interactions Practices had to engage in compensatory efforts to combat the deficits of digitisation (eg, running online and telephone booking systems was necessary to compensate for the challenges of digitisation) only for some groups but had potential to overwhelm practices	“What they say in those opening 20 to 30 seconds that is so important and matters to them all gets lost if it comes through electronic prefilters, other allied health professionals who are acting on your behalf. And it diminishes the relationship if you become remote and distant; you’re no longer someone who’s easily accessible.” (GP, 3955) “If you can just book online then people book the wrong appointments in with the wrong things, and then you end up with more of a muddle. Booking online is not great, you know. Here’s a smear clinic and then somebody books stomach ache into it . . . people just see an appointment.” (Practice manager, 854e) “I find I make mistakes if I use an app, I can’t use a smartphone. I’ve got eye, hand coordination issues. I’ve got a really bad astigmatism, and it’s very small on the phone.” (Patient, E8)

AI= artificial intelligence; GP=general practitioner.

**Table 3:** Policy shifts, study findings, and illustrative quotations: hospital to community

Key elements of policy shift	Study findings	Illustrative quotations
Shift patterns of health spending from hospitals to communities Establish a neighbourhood health centre**—**a so called one stop shop for patient care and centre for multidisciplinary teams**—**in every community that brings services together and is open 12 hours a day, six days a week Support greater multidisciplinary team working across neighbourhood areas Transition community pharmacy towards more clinical services Train thousands more GPs, increase the proportion of staff trained for community and primary care roles, and scale new community roles Cut bureaucracy in general practice Introduce a single neighbourhood provider contract to provide additional services (mainly from general practice) across areas of 50 000 patients Introduce a multineighbourhood provider contract that provides support (eg, quality improvement, consolidated back office, data analytics) to GPs and others across areas of >250 000 patients Enable foundation trusts to become Integrated Health Organisations holding whole population health budgets Review how patient need is reflected in the funding formula for general practice	Practical challenges, including patient willingness and ability to travel and practice estate limitations were seen as constraints on neighbourhood-style services Practices perceived that they would always have a key role in coordinating care for patients and be the default front door for patients, regardless of the service needed and new service configurations Transferring services out of practices to central hubs that offered additional access after core hours or to additional services (eg, long acting contraception, ear syringing) introduced additional bureaucracy and barriers for patients as well as increased friction for practices Patients perceived a lower threshold and shorter wait times for appointments with allied healthcare professionals but a higher threshold for appointments with a GP Patients with undifferentiated symptoms could often not determine which, if any, allied healthcare professional would be best placed to see them, or they worried that allied healthcare professionals would not assess them as comprehensively as a GP The potential of allied healthcare professionals to take pressure off GPs was not always fulfilled because many allied healthcare professionals recommended patients to attend a GP anyway, potentially risking duplicated visits, delays, and failure demand Operational systems for coordinating community based case was frequently suboptimal Larger practice models did not resolve the impact of the lack of GPs on access	“We haven’t got any space; we’ve taken on another GP (GP) who starts in November, but we can only start him on two days because we haven’t got the room for him to do anymore.” (Practice manager, 7779) “We have access to extended hours but those appointments are within our group, there’s quite a few of us vying for those appointments, they are quite limited and some people aren’t able to travel to those places because they’re too difficult to get to.” (Receptionist, d125) “They could go to a nearby village for nurse appointments. But some of them prefer to come here because it’s their GP.” (Practice manager, 58e6) “The patients always come to us. It doesn’t matter about all the 101 other services that get brought in, if they’ve got an issue, they’ll come to us and expect us to sort it out . . . from the patients’ point of view, we are the front door to the NHS and they expect us to be able to sort out everything.” (Practice manager, faff) “The longitudinal relationship between individual patients is the bit that allows you to do so much more with so much less.” (GP, 3955) “If you’ve got a patient that knows you and trusts you, they tend to trust what you tell them the first time round.” (GP, 4001) “We amalgamated with [practice], I think they’ve got about 20 doctors there. They're all on the noticeboard. I haven't been assigned a doctor . . . that one I saw, I’d never seen her before. I don’t even know what they were called. It’s very impersonal now.” (Patient, efe5)

GP=general practitioner.

**Table 4:** Policy shifts, study findings, and illustrative quotations: sickness to prevention

Key elements of policy shift	Study findings	Illustrative quotations
Deliver prevention at neighbourhood level using genomic technologies, diagnostics, predictive analytics Trial new prevention accelerators that deliver community led methods of improving of uptake of diabetes and cardiovascular disease interventions	Practices were already working on the prevention agenda; many had sophisticated processes for ensuring eligible patients were invited for primary and secondary prevention Prevention activities required considerable administrative resources and consumed a large proportion of appointments in practices; prevention reviews were generally longer than acute care appointments Not all patients accepted invitations from practices to engage with preventive care, although some saw it as a means to getting a face-to-face consultation that was otherwise difficult to secure Prevention generated additional downstream work for practices including questions from patients who found themselves crossing the boundary between health and illness because of prevention reviews Patient experience of prevention reviews was mixed: some found the reviews transactional rather than holistic or patient centred, and they could lead to fragmented care for patients with multimorbidity Practices’ ability to deliver high quality prevention was hampered by their lack of access to auxiliary services Clinical staff believed that much prevention work related to social determinants that they had little power to change	“Ten to 15 minutes is just the normal slot, for things like asthma reviews, things like that, they have thirty minutes, health checks, they will have twenty minutes, dementia reviews, they have a lot longer.” (Receptionist, ad11) “It’s the doctor's choice to say well, I’d rather you came in, which doesn't really often happen. As I say, the only times is when it's the review for the year. A yearly review.” (Patient and carer, 66e1) “I saw somebody who is 56, he’s not very old, but he’s got quite bad COPD . . . And we’ve had a real problem recently with no pulmonary rehab . . . And I think if we had the pulmonary rehab, it would be a lot better - normally you’d do a yearly review with COPD [chronic obstructive pulmonary disease] if they’re well, he’s been seen three times a year this last year.” (Nurse practitioner, 2328)

The plan lacks detail on how the three shifts will be delivered in practice,^12^ ^13^ so generating evidence to inform design, planning, and implementation is essential. High quality evidence is particularly important given the role of effective early planning in the success of major programmes,^14^ ^15^ the consequences of previous NHS policy initiatives with similar objectives,^16^ ^17^ and the need to engage with the views of staff and patients.^18^ We report selected findings from a large qualitative study examining recent patient and staff experiences of access to general practice, conducted at a time the system was already changing in the direction of the shifts proposed in the 10 year plan for England. This study focuses specifically on findings that provide insights into the potential effects of the three shifts for people’s experiences of access to healthcare. 

## Methods

We conducted a qualitative study using semi-structured interviews to explore recent experiences of access to NHS general practice among patients, carers, GPs, and other practice staff. Five researchers conducted the interviews (three health service researchers and two clinical researchers) between July and October 2023 using semi-structured interview guides. Each interview was recorded, transcribed, and anonymised before analysis. While data collection was underway, we discussed each interview at weekly meetings, allowing iterative refinement of the interview guides and principles of information power to guide sampling practices.^19^

### Patients’ and carers’ recruitment and data collection

Healthwatch, a statutorily independent organisation that acts as a champion for people who use health and social care, and that has branches in every local authority area in England, supported recruitment of patients and carers to interviews. Healthwatch recruitment strategies emphasised the inclusion of a diverse range of people for age, sex, ethnicity, employment status, and medical characteristics across five geographically dispersed areas in England. Community centre activities (eg, weekly walks, games sessions, and coffee mornings), social prescribers (professionals who act as link workers to connect people to activities, services, and groups in their community), and Healthwatch’s engagement with specific vulnerable groups (including people with visual impairment, learning disabilities, and migrant people) were all used as part of recruitment efforts. Participants were offered a choice of online, telephone, or in person interviews. As advised by the patient and public involvement panel for the project, each participant was offered a £25 (€28; $33) shopping voucher for their involvement in the study.

Using a semi-structured guide, interviewers asked patients and carers to narrate a recent experience of seeking a consultation or service at their general practice. Interviewers prompted patients to give a chronological description of events from when they identified their healthcare need, describing the process of getting the appointment, any barriers encountered, interactions with healthcare professionals, and their overall experience of accessing healthcare. Interviewers also encouraged participants to describe any contrasting experiences with access.

### General practice staff recruitment and data collection

General practice staff were recruited through the east of England branch of the NIHR Research Delivery Network (known as the NIHR Clinical Research Network at the time of the study). Interested practices submitted details on their practice size, location, and, as an indicator of the socioeconomic status of the population served, an estimate of local deprivation. Practices were specifically selected from this group using criteria regarding deprivation levels, practice location (urban or rural), and practice size to ensure diversity within the sample. All staff, including GPs, nurses, practice managers, administrative staff, and allied health professionals were eligible to participate, up to a maximum of four staff in each practice. Practices were remunerated using Clinical Research Network rates.^20^

For clinical staff, we used chart stimulated recall, a technique whereby clinicians are asked to describe a clinical encounter using the patient’s notes to prompt their recollection of events. The principle of this technique is that it enables a more detailed recollection than using clinical notes or the participant’s account alone, and allows interviewers to probe certain events and decisions.^21^ We asked clinicians to describe three appointments with patients (without patient identifiers) from their most recent half day of clinical practice, selecting the first, middle, and last patient on the list. Clinicians used the patients’ electronic health records to support their recall of the appointment—for example, the patient’s reason for attending and the patient’s route to securing the appointment. Patient-facing administrative staff (eg, receptionists) were asked to record, using bullet points, all requests for appointments (without patient identifiers) during a 20 minute time frame on the morning or day before the interview, and to recount these interactions during the interview. For administrative staff who were not patient-facing (eg, practice managers), a separate interview guide was used, covering different stages of patients’ access journey and any efforts they had taken or were considering taking as a practice to improve access.

### Analysis

We based our analysis on the constant comparative method.^22^ Firstly, four researchers individually coded four transcripts (two patient or carer transcripts and two practice staff transcripts), drawing on sensitising concepts^22^ (concepts that help in understanding and interpreting qualitative data) from the academic literature to support identification of themes relevant to access to primary care. Concepts included those from the Candidacy Framework,^23^ which offers insights into how people perceive their eligibility for care, how they interact with services, and how services shape need, demand, and response given their operating conditions. We also used concepts from Starfield’s 4Cs framework for high performing primary care (first contact, continuity, comprehensiveness, and coordination)^3^ and literature on multimorbidity and failure demand (demand caused by a failure to do something or do something right for a patient, generating additional work that could have been avoided if earlier contact had better met perceived patient needs).^24^ Guided by sensitising concepts, coders also undertook line-by-line coding to interrogate the data, for example to understand processes and their consequences. The coders shared their findings and developed a guide to subsequent coding. Three researchers coded all remaining interviews using Nvivo software. They captured as much context as possible in the label given to a section of text, and maintained memos on interesting, novel, or divergent findings. The team met weekly as analysis proceeded, and the coding guide was refined as additional questions arose.

The final codebook, which comprised more than 3100 individual codes, was sorted into a set of overarching themes aligning with different stages and dimensions of access journeys. These data were axial coded and synthesised into subthemes.^22^ Once completed, we wrote data summaries with illustrative quotations for each subtheme that were collated in a single document for further specification and synthesis. and then mapped to and interpreted in the context of relevant proposals in the 10 year plan. 

### Patient and public involvement

Patients and members of the public were involved in the design of our research from the outset. The patient and public involvement panel at Cambridge University Hospitals NHS Trust reviewed invitation letters, participant information sheets, and consent forms. Panel feedback led to language amendments to meet the needs of our target participants, and to the design of data collection methods that met diverse needs. We worked closely with Healthwatch, which gave feedback on patient recruitment and communication strategies, as well as facilitating recruitment of underserved groups.

## Results

We conducted 70 semi-structured qualitative interviews with a diverse mix of patients, carers, and staff (table 1). The 41 patients and carers (27 women, 14 men) interviewed were from the north west, south west, south east, and east of England. The patient and carer sample included 12 different ethnic groups and people with vulnerabilities resulting from learning disabilities, visual impairment, recent immigration to the UK, or people who were non-native English speakers. In total, we interviewed 29 general practice staff from 13 practices across the east of England, including 10 GPs, five nurses, one pharmacist, one wellbeing coach, seven practice managers, and five administrators. These practices reported having from 5001 to more than 30 000 registered patients. Two practices were part of a larger practice cluster. General practice staff had worked at their practices between one and more than 10 years. Seven practices were in more urban areas, six in more rural areas. Based on the index of multiple deprivation levels of practice postcodes, nine practices were in more affluent areas and four in more deprived areas.

**Table 1:** Characteristics of patients, carers, and general practice staff participants

Characteristic	No (%)	
	Patients (n=41)	GP staff (n=29)
**Sex**		
Male	14 (34)	6 (21)
Female	27 (66)	22 (76)
No response	—	1 (3)
**Age (years)**		
18-29	5 (12)	3 (10)
30-39	3 (7)	5 (17)
40-49	5 (12)	10 (34)
50-59	13 (32)	10 (34)
60-69	5 (12)	1 (3)
70-79	6 (15)	—
≥80	4 (10)	—
Ethnic group*		
White	30 (72)	22 (76)
Mixed or multiple ethnic groups	1 (2)	2 (7)
Asian or Asian British	5 (11)	3 (10)
Black, black British, Caribbean, or African	3 (6)	—
Other	2 (4)	1 (3)
No response	—	1 (3)
**Employment status**		
Full time paid work (≥30 hours weekly)	12 (29)	—
Part time paid work (<30 hours weekly)	6 (15)	—
Permanently sick or disabled	3 (7)	—
Retired	10 (24)	—
Unemployed	6 (15)	—
Other (volunteer/self-employed)	4 (10)	—
**Location of practice**		
East of England	12 (29)	—
North west	14 (34)	—
South east	6 (15)	—
South west	8 (20)	—
Other	1 (2)	—
**Length of time working in GP setting (years)**		
1-5	—	8 (28)
5-10	—	4 (14)
>10	—	16 (55)
No response	—	1 (3)
**GP role**		
General practitioner	—	10 (34)
Nurse	—	5 (17)
Receptionist or administrator	—	5 (17)
Practice manager	—	7 (24)
Health and wellbeing coach, pharmacist	—	2 (6)
**Size of general practice (No of patients)**		
5001-10 000	—	8 (28)
10 001-29 999	—	20 (69)
>30 000	—	1 (3)
**Rural versus urban GP location (n=13)**		
Urban	—	7 (54)
Rural	—	6 (46)
**Deprivation level of GP setting (n=13)**		
More affluent than deprived	—	9 (69)
More deprived than affluent	—	4 (31)

GP=general practice.

* Grouped by Office for National Statistics categories.

We have organised our findings around three key themes of the 10 year plan’s proposals that are relevant to primary care access: the shift from analogue to digital, hospital to community, and sickness to prevention. Table 2, table 3, and table 4 summarise these shifts and our main findings along with illustrative quotations.

### The shift to digital

The 10 year plan strongly emphasises digitising many aspects of primary care (table 2) as part of the solution to frustrations with access to care. At the time of our study, the shift to digital access was already being strongly encouraged at policy level and was at any advanced stage in many practices. We identified mixed impacts of the shift to digital: while it was recognised as offering some benefits, digitisation was not seen as a solution to the fundamental lack of capacity in general practice. Digitisation was perceived to introduce new forms of burdens of access with inequitable impacts, and to increase duties of appointment stewardship for practices.

#### Digitisation and the limits of capacity

Staff and patients both described how appointment booking systems were the most evident target of access digitisation in general practice, often displacing older first come, first served telephone queuing systems. These telephone systems had limited how many patients or carers could successfully contact their practice during a defined time slot, and were often frustrating for patients, stressful for staff, and sometimes perceived as unsafe. Digitisation was therefore recognised as offering potential benefits: some participants described appointments they made by booking directly online (without having to go through triage at the practice before confirmation). More generally, however, a move to a full self-service model, whereby patients could simply book the appointment they wanted and when they wanted it, was largely seen as unfeasible by staff: the practice lacked the capacity to facilitate it, and matching patient need to the right type of appointment was seen as complex. Staff emphasised that digital systems alone did little to resolve the fundamental mismatch between the limited number of and high demand for appointments (particularly with GPs).

“You always feel there still aren’t enough appointments or there are sometimes . . . for my own clinics, there are quite long waits to be seen if they want to see you specifically.” (GP, 2c62)

To manage demand, practices typically used specialised software that, after asking patients to describe the nature of their request (eg, symptoms and expectations), triaged incoming requests. Triage might result in a range of outcomes, only one of which was an appointment with a GP (and when such an appointment was offered, it might be based remotely rather than in person). Practices varied in their extent of digital transformation, with some continuing to run telephone systems and digital systems simultaneously.

#### Benefits and burdens of digitisation of access

Patient and staff response to digitisation was mixed: some valued the perceived efficiencies and convenience of digitally enabled access to primary care.

“We encourage people to use the NHS app, [. . .] something we’ve really pushed this year and we stopped phone access, we stopped email access, only take phone access from truly housebound people. Other than that, it has to be via the app or a paper slip . . . we’ve pushed that really hard and we’ve had sessions, enabling sessions to help people get on the app and things like that.” (Administrator, 99c3)

“Now appointments, everything has been made online . . . I think that is a good step. It will save a lot of time in making appointments.” (Patient, 1cfe)

However, digitisation was not seen as a straightforward solution to improving access. The shift to digitised care (eg, more online form filling, more triage and appointments by telephone) often required additional contact points for patients compared with a single call for an appointment and then seeing a clinician face-to-face. While patients gained some forms of control, they also lost other forms of agency—for example, having to wait for a return phone call. Any mistake or lack of clarity in digital communication created more uncertainty and further need for contacts. Digitisation was also repeatedly identified in interviews as contributing to inequities in access. One challenge was that making bookings more convenient was seen by some staff to increase demand by lowering some patients’ threshold for requesting an appointment or advice, potentially consuming resource and creating opportunity costs (loss of options to do other things with the resource).

“And sometimes in the day and age of social media, instant access that patients perhaps are a bit too trigger happy at contacting.” (Pharmacist, 5e3e)

Although digitisation eased access for some groups of patients, the shift created, for other groups, what might be termed a burden of access problem (a component of the previously described concept of burden of treatment).^25^ Here, the workload, effort, and assets required to access care increased for some patients, particularly vulnerable patients. Patients and staff recognised that digitisation required resources that were unevenly available, especially for people who were socioeconomically disadvantaged or older, or those who had a disability, impairment, or frailty.

“We shouldn’t switch off all access because it’s just not fair. Sometimes people just come in, and they have maybe 10 minutes with admin because it’s actually they can’t get this sorted, or that sorted, you know. Prescriptions are online, everything’s online [. . . ] it sounds like utopia, but we are concerned about the amount of people . . . I think it’s easy to lose sight of the amount of people that can’t do these things. We all move to a world where everyone can do everything online in our heads, and we forget.” (Practice manager, 854e)

“I’d like to think I can articulate well verbally and in writing, it wasn’t an issue for me. But it would be an issue for many others.” (Patient, K2)

Some of the burdens were logistical: some patients could not book appointments because they did not have or could not use the necessary devices. Other burdens were linked to cognitive, linguistic, and social capabilities: some patients could not give an account of their needs through prestructured forms, or struggled with asynchronous text or email messages, leading to missed treatment, appointments, and other care.

### The shift to community

The 10 year plan proposes introduction of neighbourhood based care models intended to harness economies of scale by sharing resources and achieving efficiencies by increasing the scale of operation and to deliver additional services in larger geographical areas (table 3). Some elements of this kind of shift were already underway at the time of our study, including an expanded mix of professional skills within practices, hub models staffed by multidisciplinary teams providing extra appointments (often for same day care), and offering services across wider areas. Again, we found mixed responses.

#### Expanding the options available for care outside single practice boundaries

Staff and patients expressed some positive comments on expanding the range of options, including multi-disciplinary teams based in hubs, for those willing to travel outside of single-practice boundaries.

“There’s a minor illness nurse that works in the new hub in town and she takes the overflow for the surgeries, if somebody needs a minor illness kind of thing and we’ve got no appointments on the day, we can just book them down there.” (Practice manager, 85d5)

“[The access hub] gives them the option to not have to worry about missing out on work, so not missing out on pay, and things like that, is definitely a bonus for them. You know, if it means that they don’t have to worry about waiting, say, sort of, three or four weeks, they can be seen within the week, to get a blood test done. Yeah, definitely, definitely, a big pro.” (Receptionist, edab)

Neighbourhood-like models did not, however, appear to offer straightforward solutions to improving access and people’s satisfaction with access. One practical problem was that constraints on space within practices were already, at the time of study, limiting what was possible. Expanding the access options for patients, including different locations and wider staff roles, was seen to generate extra work for practices, including organisational and coordination demands, as well as the need to manage patient expectations. Another challenge was that patients tended to see their practice as the default for accessing health services, and they were not always willing or able to be diverted to an unfamiliar setting or to be confronted with costs and logistical challenges.

“And one of the practice managers said, well, we have subscribed to the hub. I don’t know what the hub means, I said, I don’t want to see anybody in the hub.” (Patient, 8160)

“Well we would prefer [services] to be in our GP’s surgery, because otherwise we have to incur more costs to get to them.” (Patient, JW1)

Where aspects of neighbourhood based care would be desirable, we found they would require active design and management. Staff reported lack of integration and coherence between services, since general practice was seen as the so called front door to the wider health service by patients, but practices lacked authority and mechanisms to efficiently coordinate or facilitate care outside their own boundaries. GPs were also exasperated by their inability to arrange key investigations or to provide access to therapeutic services directly.

“I’m not allowed to get my own echocardiogram, I can’t, I’m blocked from requesting it directly, I used to be able to do that. That’s important because it used to confirm the diagnosis for me while waiting and it meant I could refer them to the community heart failure nurses, extra support in the community.” (GP, 4eae)

Similarly, patients complained of difficulties with care coordination and coherence—for example, when trying to find out where referrals to external services were in the system. Where changes had been made to manage services at supra-practice or network level, extra steps were sometimes needed to complete the same tasks. For example, patients described needing GP referrals for services such as ear syringing and podiatry that they had previously been able to arrange directly at their practice. If managed at network level, two transactions (appointment with a GP which was hard to get, for a referral, and then attend the external location for the service) were required; previously only one was needed. When patients did use services outside of the practice, they believed that their GP should maintain oversight of their care.

“He was referred to mental health services through the drug and alcohol services, because he's drinking . . . because he's got a mental health problem that's not been seen to, he turns to drink to cope, and he was having help for that. They have referred him to the mental health services, so it never went to the GP. So, a GP has never . . . normally your GP should understand your care and we felt that at this big practice they didn’t.” (Patient and carer, E9)

#### Diversification of mix of skills

A greater mix of professionals and new roles within practices, of the type now anticipated to be scaled up at neighbourhood level, had led to more appointments in many practices at the time of our study, typically with members of the primary care team other than GPs. Responses were often positive about individual staff.

“The physio was lovely, he was very understanding. I had a good appointment with him and he gave me some exercises and he booked me in for a follow up appointment at a time that I could choose, which was really nice.” (Patient, 3637)

Although patients valued the expertise of individual professionals for particular health problems, they expressed resistance to the idea that the only thing that mattered was getting an appointment, regardless of whom the appointment was with. In general, both clinicians and patients highly valued a personal, longitudinal relationship with a known GP (or other health professional), characterised by continuity of care.

“I would like to deal with somebody that knows me, not only knows the condition I'm talking about and other conditions I might have, but understands the sort of person I am [. . . ] And I would much rather have somebody that I can speak to and deal with and that they can ask me an open question, anything they like, and it would be an honest conversation between the two of us.” (Patient, E6)

“It’s better when it’s the same person because I’m immediately aware of why I’m seeing them. I don’t have to trawl through the notes to, sort of, double check quite where were we at. And it gives you continuity, it means I know what I’ve tried, or what I was thinking and it’s, sort of, a clear pathway for them.” (GP, 4eae)

In contrast, participants reported that treating professionals as interchangeable or configuring appointments as a bundle of tasks to be completed could cause confusion, fragmentation, inefficiencies, and transactional relationships. Even if patients had been given an appointment, they did not always feel that they had achieved access if the appointment did not meet their needs. They reported being frustrated by seeing professionals with more limited skillsets and narrower approaches to clinical management. Patients who had expected to see a GP but were allocated to allied health professionals sometimes reported that they did not always fully disclose their problems or trust the advice given. Sometimes, they refused the appointment or perceived their consultation as unsatisfactory, with some seeking to re-attend to see a GP.

“My only concern with seeing the physio is he’s only looking at the knee in isolation, whereas I wanted to see a GP that would look at my health as an overall picture.” (Patient, 3637)

Clinicians reported that one well coordinated appointment led by an experienced GP was often the most efficient, satisfying, and effective way of managing patients’ problems, particularly when part of a longitudinal relationship. They emphasised how professionals’ intimate knowledge of how a patient ‘normally presents’ could sensitise them to potentially undisclosed symptoms or changes in the patient over time, increase the efficiency of consultations, or provide valuable context for what had previously been tried. Allocating this work to others, however, was seen as risking undermining essential relational work and holistic care.

“That time when you wanted to see your GP, and they were on leave, and they offered for you to see a nurse, why did you feel uncomfortable seeing a nurse? R: Because I didn’t know who they were. Like I say, because [GP] understands, he knows what I’ve been going through with my own mental health. I don’t want to have to keep explaining myself to other nurses and other doctors, for them to pass me on to someone else and someone else, ’cause I’ve gone through that situation before.” (Patient, JW4)

“Concentrating on continuity of care because you will retain more GPs with that because it makes the job more enjoyable when you know the patients, it means that you can actually deal with it in 10 minutes because you know the patient rather than getting somebody that you’ve never seen before and yet they have to give you the back story, they know that you don’t know them so they want you to know. That makes appointments a lot longer than they would be if you did know the patient.” (GP, 4001)

### The shift to prevention

The 10 year plan prioritises a shift from treating to preventing illness, including early detection and intervention. Primary care is expected to intensify its efforts and to address variation in the uptake of both primary prevention (eg, vaccination, lifestyle support for smoking reduction, diets, and exercise) and secondary prevention (eg, managing long term conditions and risks of cardiovascular disease and diabetes). Similar to the other two shifts, our study suggests the perceived benefits of such an approach may be mixed.

#### Burden of prevention

In our study, practices were already demonstrating strong engagement with the prevention agenda, influenced in part by financial incentives such as the Quality and Outcomes Framework. However, the organisation of preventive care (including interventions aimed at primary prevention, such as vaccination, and those aimed at secondary prevention, such as chronic disease management) involved substantial administrative work. For example, practice staff had to establish and maintain up to date registers of patients who might be eligible for interventions, repeatedly notify patients of their eligibility, organise appointments for blood tests and other investigations, arrange reviews once all test results are available, check that patients had attended their reviews, ensure that the reviews had been completed in accordance with set protocols, and maintain records of making claims for payments due to the practice for these activities. Considerable effort was required to fill some preventive appointments, since some patients did not accept them if they felt well, even when approached proactively by staff.

“We do all the recalls for the patients, making sure they get in, come in and then my part of that is claiming for everything that is done in the surgery . . . we have to get all that information in, complete and submit all the forms . . . The girls will have been continuing to hit targets that we have to reach but there are seven of us on our team here and we are pretty good at hitting the targets and getting all the patients in . . . it will be a lot of ringing around, a lot of checking that patients have been in, had their reviews, they’ve been done properly and they’re covered for another year.” (Receptionist, ad11)

Clinical staff also reported that, despite the prominence of the prevention agenda for primary care, much preventable ill health related to health behaviours and social determinants such as housing and education, factors that they had little power to change.

“Ninety to 95 per cent of the stuff that we see is lifestyle. If they didn’t drink alcohol, if they didn’t smoke, if they ate healthily, if they did some exercise, we wouldn’t see them . . . So, it’s actually a really complex issue as to how do we address this.” (GP, 4001)

#### Patient experience of prevention in general practice

Many patients with a long term condition did value care reviews, but this was often because they struggled to get appointments at their own request, and the reviews gave a rare opportunity to be seen in the practice in person. However, they also reported that reviews sometimes felt highly transactional, generic, and dominated by task focused clinical agendas, not as patient centred care. A particular concern reported by patients was the balance between mandated prevention activities and the ability of practices to respond to patient needs, which some patients saw as undermining prevention as they conceived it.

“I didn’t get a proper annual review. I had one, but it was just one nurse, and she did one blood test for one thing and took my blood pressure . . . no discussion, I couldn’t talk about anything that was bothering me that I did have symptoms of, and it just didn’t seem to me that it was a proper review.” (Patient, E8)

“Well she does the blood pressure first, then she does the bloods, and then she does the weighing, and that’s it really. You just get thrown out the door and you think, well why am I here?” (Patient, E5)

Similar to the consequences of the shift to community, the so called taskification of secondary prevention, including chronic disease management, was perceived to increase fragmentation of care. Patients with multiple conditions often saw different healthcare professionals for each condition, rather than one who could address all their conditions holistically. Approaches to clinical management were not always coherent, resulting in frustration and safety issues, for example, in relation to drug prescribing.

“I had a text from the surgery to book an appointment for an asthma review, a diabetic check and foot check and obviously had my bloods taken. I had four separate texts and one for the flu and covid vaccine. So I had four separate texts come through.” (Patient, cadc)

## Discussion

### Principal findings

Our study, involving 70 interviews with patients, carers, and healthcare staff suggests that the three shifts sought by the government’s new 10 year plan^10^—to digital, to community, and to prevention—may have mixed impacts on experiences of access to general practice care in England.

We found that the digitisation of access systems, including a concerted effort to transition away from traditional telephone-based appointment booking systems, has been underway for some time. Study participants recognised innovations such as online booking and triage systems as offering increased efficiency and convenience for some. However, they were also perceived as systematically favouring people with digital literacy and good digital access, while introducing additional forms of disadvantage and exclusion, especially for patients with vulnerabilities. Staff and patients expressed concerns about possible de-humanisation associated with digital communication—for example, reduced ability to understand patients’ needs or identify potential disadvantages or vulnerabilities. Practices described efforts to mitigate these issues, including tailored arrangements for disadvantaged groups and personalised communication to reassure patients, but these measures were resource intensive. A fundamental challenge was that greater digital access did little to address the basic mismatch between demand for seeing a GP (ideally one known to the patient) and the number of available appointments.

Our study suggests that the shift from hospital to community, including the proposed redistribution of specialist services outside hospitals, is similarly likely to offer a mix of benefits and challenges. Features of neighbourhood based care aimed at improving service delivery through hub-like facilities, staff with an expanded mix of skills, and multidisciplinary teams were reported in our study, and they were recognised as offering more options and increasing access in some ways. However, these initiatives also posed multiple practical and logistical challenges, ranging from space constraints and complexities of coordination to patient reluctance or inability to travel. The appointments on offer did not necessarily provide the kind of access that patients sought, nor were they always effective and efficient in solving the problem at hand. Participants emphasised the importance of having a single authoritative lead clinician who could make holistic assessments and decisions. Staff and patients valued continuity of care and the relational aspects of GP-patient interactions, finding these elements to be more satisfying, more efficient, and more effective in meeting patients’ needs. Conversely, new roles and services across wider geographies were seen as increasing coordination challenges and risks of fragmentation without always offering clear efficiency gains.

We found that preventive care efforts were already extensive in general practice, and were using considerable resources in their organisation and administration. As with the other two shifts, staff and patients reported mixed experiences; for patients, reviews and other preventive interventions could feel transactional, task focused, and reductionist (ie, focused on a single disease or body part). Some patients might regard preventive appointments as non-essential and decline attendance unless they were experiencing symptoms, meaning administrative staff were trying to fill appointments for prevention and long term conditions while demand for appointments for patient initiated or acute care outweighed availability. Clinicians saw many of the wider social and economic factors affecting the health of their patients as beyond the scope of primary care.

### Strengths and limitations of the study

This study provides evidence relevant to the 10 year plan, helping to go beyond speculation about experiences of access to general practice in England. A considerable strength of our study was that several aspects of the major shifts proposed by the 10 year plan—including increased digitisation, expanding the role of allied health professionals, consolidation of services across larger geographies, and an emphasis on illness prevention—were already ongoing at the time we undertook our interviews, following a series of policy initiatives before the 10 year plan for England was published.^9^ Concern was already being publicly expressed about the potential impacts of the proposed shifts prior to their inclusion in the plan^26^ ^27^ ^28^—such as digitisation potentially widening inequalities—and this concern is reflected in many of our findings. Multiple aspects of context have remained remarkably similar over the period since the interviews were conducted. For example, NHS data on appointments in general practice show that they have remained largely unchanged since then in terms of number of appointments on offer, timeliness, and proportions of appointments with GPs versus other clinicians.^29^ Our study, therefore, provides insight into a post-covid-19 pandemic era that already featured many of the key policy directions of the 10 year plan, with many aspects of context remaining constant. Our results may therefore play a valuable role in anticipating and managing the anticipated impacts of the plan.

Other strengths of our study were the inclusion of patients and carers across England with a diverse range of personal and medical characteristics. Our interviews with staff focused on only one region of England for reasons of practicality and resources, but we were able to include staff with varied role responsibilities and levels of experience, practices of different sizes, and locations serving populations with varying levels of socioeconomic status.

In the context of a wide range of experiences of access in general practice, our study was enhanced through iterative refinement of interview guides and purposive sampling, which were in turn enabled by concurrent interviewing and analysis.

It is possible that recruitment of patient and carer participants through Healthwatch may have led to overrepresentation of people who were particularly engaged with healthcare services. However, by asking participants to discuss very recent interactions we gained insights into everyday care, not just outliers and extreme examples. Decoupling patient and professional experiences of the same interactions was a study design choice to enable participants to speak more freely, but it did limit the insights that might otherwise have been available—for example, by enabling direct comparison of experiences in pairs of staff and patients. As we have only reported aspects of our larger study relevant to the 10 year plan, we have also not explored the full range of influences on or experiences of access to general practice.

 Some evidence suggests that experiences of access vary across the different regions of the UK. For instance, recent surveys show that more people in Scotland (50%) than England (43%) find it very easy or easy to have contact with their general practice.^30^ Interpretation of these differences is not straightforward, not least because the devolved nature of healthcare policy has resulted in highly variable systems and structures, and our study did not collect qualitative data beyond England. However, it is notable that Scotland, with its higher percentage of those reporting ease of access, has more GPs per person,^31^ ^32^ and that the new Scottish GP contract in 2018 emphasised holistic and person‑centred rather than disease centred care, care of patients with complex needs, acting to reduce health inequalities, and retiring the Quality and Outcomes Framework.^33^ International comparisons are, of course, confounded by differences in the structure and role of primary care across health systems and, depending on the measures used, the tendency of the NHS to score highly on access because it is free at the point of use, in contrast to many other countries.^34^ Nonetheless, primary care services across countries face common challenges, such as workforce pressures and managing increasing numbers of people with multimorbidity. Several countries are developing common strategies in response, such as more team based models of care.^35^ Future research might seek more in depth analysis of variations and experiences of access within the UK and internationally.

### Literature comparison and policy implications

Our study affirms that people have preferences relating to different dimensions of access to general practice, including choice of clinician, type of healthcare professional, and mode of consultation. Patients also value having a nearby practice, easy booking systems, short waiting times, continuity of care,^37^ ^38^ ^39^ and being kept informed.^36^ They appreciate these dimensions differently depending on age, morbidities, and other characteristics, and their ability to access care is shaped by a range of personal, social, and institutional forces.^40^ Previous efforts by national policymakers to improve access to general practice in the NHS have been plentiful,^9^ but they have often been narrowly focused on availability of appointments and typically underplay the other dimensions of care that matter to patients. Our findings suggest that the 10 year plan is at risk of repeating some earlier failings, and that none of the three shifts (digital, community, or prevention) will straightforwardly deliver the improvements in experiences of access that patients seek.

By enabling patients to perform tasks once undertaken by administrative or clinical staff, the plan envisages digital transformations similar to those in other industries, such as banking, travel, and entertainment, as noted by the prime minister at the launch of the plan.^41^ The rise of self-service models in these other sectors has been driven by convenience and improved user experience for customers, and by cost reduction and efficiency for businesses.^42^ Our study suggests that, in the NHS in England, achieving such a transformation in full is unlikely to be possible if the NHS founding principle of universal access on the basis of need is to be preserved. Since digitised booking of appointments requires inequitably distributed resources, including both technical facilities and ability to articulate a problem that can be clinically recognised and processed, it raises the risk that some patients’ needs might go unrecognised or unmet. Digital convenience could induce demand by some and could further exclude the needs of people who cannot use these technologies.

Our study emphasises that reconfiguring patients as digital candidates^43^ for care requires understanding the nature of the work asked of patients, how they do it, and the recognition that this work is much easier for some than for others. Unwarranted variation in access to GPs is already problematic and persistent,^44^ and it may well be compounded by digitisation^43^ ^45^ ^46^ as burden of access becomes an increasingly prominent part of burden of treatment. The 10 year plan does acknowledge the risk of digital exclusion, but proposes to address it primarily through the design of the NHS app and provision of additional support for those at risk of exclusion. This strategy fails to acknowledge how inequalities in access happen through the complex interaction of patients, technology, staff, and wider social systems in which they operate. Consistent with previous studies of digital first primary care,^47^ our study indicates that digitisation, while likely to deliver some benefits, will require major design investment, recognition of the distinctive nature of healthcare, and sensitivity to inequities in care access.

Digitisation also does little to address the mismatch between what many patients most wanted—an appointment with a GP, and ideally one known to them—and what they can access. The context is one where demand for appointments has risen, but the number of fully qualified, full time equivalent GPs has decreased since 2015.^48^ ^49^ The number of appointments with a GP is, in operations management terms, a classic bottleneck: the resource with the greatest impact on overall system performance.^50^ Improving process efficiency (how easily an appointment can be made through a platform) will result in little marginal gain if the bottleneck remains. While the plan seeks to “bring back the family doctor”,^10^ it is vague on what that means in practice. Matching demand for GP appointments with availability is unlikely to be possible in the short to medium term, even with the training of extra GPs promised in the plan. The mismatch between demand and availability might partly explain why job satisfaction among current GPs is low compared to other countries, and why many GPs plan to leave patient care or reduce their hours.^51^ ^52^

To reconcile the lack of GPs with the increasing demand for appointments with them, the broad goal of the neighbourhood model appears to be that GPs working together at greater scale over larger geographies and with a wider mix of staff could boost access to care. Our findings highlight some of the tensions associated with this shift. Expanding the range of professionals and locations for accessing primary care could increase the number of appointments available to patients. However, increases in the number of appointments in primary care over the last five years has coincided with low levels of public satisfaction, suggesting that it is not simply getting an appointment that matters, but the ability of the patient to secure the right type of appointment.^48^ Nor is satisfaction with access all about speed; around half of appointments are already booked on the same day.^29^

Our study suggests some reasons for the apparent discrepancy between increase in appointments and persistently low patient satisfaction: patients may be seeking care, social connection, and a sense of being valued. Patients and clinicians both value a personal, longitudinal relationship with a known GP. Strong evidence suggests that such continuity of care is associated with fewer patient complaints,^53^ reduced mortality, fewer hospital admissions, and fewer emergency department visits.^54^ However, larger practices are associated with lower continuity of care between a given clinician and patient,^55^ ^56^ ^57^ and it is possible that the larger footprints of neighbourhood models will have similar effects on continuity. Multidisciplinary teamworking in primary care is not new and, while it offers a mix of potential benefits for patients and staff, the desired outcomes are not always delivered and implementation is challenging.^58^ ^59^ ^60^ Organising care over larger areas may also have environmental impacts—for example, associated with staff and patients having to travel further to access care.

These findings help to identify some of the possible impacts of the 10 year plan’s policies on demand for appointments. While some drivers of rising demand are demographic,^61^ some are system generated. A key risk is that the plan continues to prioritise speed of access over relational and care continuity.^37^ ^38^ ^39^ The consequence for access over the long term may be high: relational continuity is associated with substantially longer intervals to next consultation (about 18% longer for patients seeing their most frequently consulted GP).^62^ ^63^ The effect sizes are large, suggesting efforts to improve access by mainly increasing the number of available appointments—regardless of which healthcare professional they are with—may paradoxically increase demand for appointments.^64^

Over recent decades, policymakers have repeatedly made neighbourhood style promises of the type made in the plan, yet the balance of spending and activity has shifted to hospitals instead,^65^ creating large sunk costs and path dependencies, where past decisions make later switching to alternatives difficult or costly and be difficult to unravel. Nor is the evidence about previous attempts to develop so called one stop shops (neighbourhood based centres) particularly encouraging. For example, a previous generation of walk in centres may have generated unwarranted demand, led to duplication (eg, of appointments), and caused confusion about where to go for care, resulting in “paying twice.” 66 Our study suggests that these issues remain salient, with general practices reporting that operating across practice boundaries, while offering some benefits, leaves them with heavy burdens of coordination and compensatory labour.^67^ Our study also shows that integrating services into a single network, such as a neighbourhood, may introduce new complexities that cause additional frustrations for practices and patients. For example, far from the promise of cutting red tape, sharing of resources (such as clinician ordered investigations) creates multiple operational interdependencies that have to be managed. Typically, this is done through gatekeeping processes that, as patients and clinicians reported in our study, increase friction and reduce efficiency.^68^ Nor are the issues likely to be resolved when patients are able to bypass general practice and use the NHS app to refer directly to some specialist services. For example, self-referral to physiotherapy does not save time as expected: patients continue to attend general practices for fit notes, imaging, diagnosis, and pain management, but self-referral may generate new demand, and run into bottleneck problems of its own without an increase in available therapists.^69^

Our study further illustrates the trade-offs involved in encouraging a greater focus on preventive interventions in an environment with limited resources. Consistent with previous research, we found that a large proportion of general practice work is already focused on primary and secondary prevention activities,^26^ ^70^ much of it incentivised through pay-for-performance or fee-for-service. Policy has also encouraged practices to go further upstream to identify and work on patients’ social needs; for instance, through social prescribing programmes to identify unmet social needs, such as food insecurity, and refer patients to non-medical support.^71^ Preventive efforts in primary care clearly have a valuable role, but, as identified by the study participants, the powerful impacts of the social determinants of health^72^ mean it is unlikely these efforts will change the demand equation any time soon. Adding further preventive interventions to general practice workload that consume available resources, while demand for what patients want is increasing and cannot be met, is likely to lead to frustration and dissatisfaction.

Finally, while improved efficiency is one of the goals of the 10 year plan, our study identifies how digitisation, role diversification, expanded geographies, and preventive efforts can create new burdens of access for patients, including the risk of multiple contacts for problems that could potentially have been resolved with one or two appointments. Treating professionals as interchangeable or appointments as a series of tasks to be completed risks confusion, fragmentation, and transactional relationships, and it is unlikely to improve satisfaction with access or efficiency.

### Conclusions

Our study suggests considerable challenges in delivering on the ambitions of the 10 year plan for improving access to general practice. At the time of our study, patients wanted a prompt, easily bookable appointment with a GP, and ideally one with whom they could have a longitudinal relationship for continuity of care. Yet this was the most scarce resource on offer. None of the solutions proposed in the plan appear to change this fundamental mismatch, and some potentially make it even more difficult for patients, particularly those most socioeconomically disadvantaged, to secure the care they want. A major flaw is that the plan has not done enough to clarify either how it benefits patients or how it can be implemented. However much the NHS app is positioned as the digital front door and whatever the commitment to training of GPs, it is likely to be impossible to end the so called 8 am scramble and restore the family doctor (both of which are stated goals of the 10 year plan) to the extent that patients and clinicians desire, which prompts the question: what constitutes reasonable alternatives? The detail and implementation of the 10 year plan will require considerable codesign and careful assessment of the impacts on help-seeking behaviour, patient experience, care coordination, equity, and outcomes, especially for those with complex care needs and those at risk of disadvantage.

**What is already known on this topic**

Access to general practice is currently a priority for patients in the NHS in England
Despite an increase in number of appointments, public satisfaction with access to NHS general practice has decreased over recent years
Improving access to general practice is a key element of the government’s new 10 year health plan

**What this study adds**

None of the three shifts proposed by government—to digital, to community, and to prevention—is likely to meaningfully affect public dissatisfaction with access to general practice
Some aspects of the three shifts are likely to increase inequities and create other unintended consequences, such as increased workload in general practice, new demand, and burden of treatment
More clarity is needed on what the benefits are to patients and what is sufficient in terms of access, along with careful codesign and evaluation of the three shifts

## Data Availability

Data are available upon reasonable request to the corresponding author. Anonymised interview transcripts are available, subject to development of a data sharing agreement with The Healthcare Improvement Studies Institute and additional ethical approval, which would bind researchers to the same ethical requirements as the original study.

## Notes

Contributors: CS, AAn, EP, JB, HA, and MDW conceived the study. CS, AAn, and EP undertook data curation and project administration. CS, AAn, EP, KH, and JW, were involved in data collection. CS, AAn, EP, KH, AAl, and MDW were involved in methodology and formal analysis. MDW acquired funding. CS, AAn, EP, HA and MDW wrote the original draft of the manuscript and all authors were involved in review and editing. CS is the guarantor. The corresponding author attests that all listed authors meet authorship criteria and that no others meeting the criteria have been omitted.

Transparency: CS (the manuscript’s guarantor) affirms that the manuscript is an honest, accurate, and transparent account of the study being reported; that no important aspects of the study have been omitted; and that any discrepancies from the study as planned (and, if relevant, registered) have been explained.

Dissemination to participants and related patient and public communities: A copy of the paper and a lay summary will be sent to participants who consented to receiving communication about the study results, as well as to Healthwatch.

Provenance and peer review: Not commissioned; externally peer reviewed.

## References

[1] Starfield B Shi L Macinko J . Contribution of primary care to health systems and health. Milbank Q 2005;83:457-502. 10.1111/j.1468-0009.2005.00409.x. 16202000 PMC2690145

[2] Kringos DS Boerma W van der Zee J Groenewegen P . Europe’s strong primary care systems are linked to better population health but also to higher health spending. Health Aff (Millwood) 2013;32:686-94. 10.1377/hlthaff.2012.1242. 23569048

[3] Starfield B . Is primary care essential? Lancet 1994;344:1129-33. 10.1016/S0140-6736(94)90634-3. 7934497

[4] Fisher R Alderwick H . The performance of general practice in the English National Health Service (NHS): an analysis using Starfield’s framework for primary care. Health Aff Sch 2024;2:qxae022. 10.1093/haschl/qxae022. 38770436 PMC11103734

[5] Nuffield Trust QualityWatch. Access to GP appointments and services: Nuffield Trust, 2024.

[6] Levene LS Baker R Walker N Williams C Wilson A Bankart J . Predicting declines in perceived relationship continuity using practice deprivation scores: a longitudinal study in primary care. Br J Gen Pract 2018;68:e420-6. 10.3399/bjgp18X696209. 29739778 PMC6002014

[7] Levene LS Baker RH Newby C Couchman EM Freeman GK . Ongoing decline in continuity with GPs in English general practices: a longitudinal study across the COVID-19 pandemic. Ann Fam Med 2024;22:301-8. 10.1370/afm.3128. 38914438 PMC11268676

[8] The Health Foundation. GP access tops list of public concerns about the NHS. https://www.health.org.uk/press-office/press-releases/gp-access-tops-list-of-public-concerns-about-the-nhs, 2025.

[9] Sinnott C Price E Ansari A What's been tried: a curated catalogue of efforts to improve access to general practice. *BJGP Open* 2025:9:BJGPO.2024.0184. 10.3399/BJGPO.2024.0184 PMC12421264 39788604

[10] Department of Health and Social Care. Fit for the future: the 10 year health plan for England, 2025.

[11] Department of Health and Social Care. Press Release: Government takes action to deliver neighbourhood health services, 9 July 2025.

[12] Alderwick H . Government’s 10 year plan for the NHS in England. BMJ 2025;390:r1396. 10.1136/bmj.r1396. 40615161 PMC12229272

[13] Klaber B Dixon-Woods M Augst C BMJ Commission on the Future of the NHS . Delivering on the 10 year health plan for England. BMJ 2025;390:r1980. 10.1136/bmj.r1980 41027708

[14] Williams T Vo H Samset K . The front-end of projects: a systematic literature review and structuring. Prod Plann Contr 2019;30:1137-69. 10.1080/09537287.2019.1594429.

[15] Williams TM Samset K Volden GH . The Front-end of Large Public Projects: Paradoxes and Ways Ahead. Taylor & Francis, 2022 10.4324/9781003257172.

[16] Tan S Mays N . Impact of initiatives to improve access to, and choice of, primary and urgent care in the England: a systematic review. Health Policy 2014;118:304-15. 10.1016/j.healthpol.2014.07.011. 25106068

[17] Lewis RQ Checkland K Durand MA . Integrated care in England–what can we learn from a decade of national pilot programmes? Int J Integr Care 2021;21:5. 10.5334/ijic.5631. 34754281 PMC8555475

[18] Martin GP Carter P Dent M . Major health service transformation and the public voice: conflict, challenge or complicity? J Health Serv Res Policy 2018;23:28-35. 10.1177/1355819617728530. 28870096 PMC5768261

[19] Malterud K Siersma VD Guassora AD . Sample Size in Qualitative Interview Studies: Guided by Information Power. Qual Health Res 2016;26:1753-60. 10.1177/1049732315617444. 26613970

[20] NIHR. Online SoECAT Guidance v.1.1 March 2023. https://www.nihr.ac.uk/online-soecat-guidance accessed 13 October 2025.

[21] Sinnott C Kelly MA Bradley CP . A scoping review of the potential for chart stimulated recall as a clinical research method. BMC Health Serv Res 2017;17:583. 10.1186/s12913-017-2539-y. 28830405 PMC5567630

[22] Charmaz K . Constructing grounded theory. Sage Publications, 2006.

[23] Dixon-Woods M Cavers D Agarwal S . Conducting a critical interpretive synthesis of the literature on access to healthcare by vulnerable groups. BMC Med Res Methodol 2006;6:35. 10.1186/1471-2288-6-35. 16872487 PMC1559637

[24] Walley P Found P Williams S . Failure demand: a concept evaluation in UK primary care. Int J Health Care Qual Assur 2019;32:21-33. 10.1108/IJHCQA-08-2017-0159 30859878

[25] Smyth RC Smith G Alexander E May CR Mair FS Gallacher KI . A systematic review of the use of burden of treatment theory. J Multimorb Comorb 2025;15:26335565251314828. 10.1177/26335565251314828. 40352785 PMC12064904

[26] Sinnott C Dixon-Woods M . Reversing the spiral of dissatisfaction in access to general practice-what can the new government do? BMJ 2024;386:q1622. 10.1136/bmj.q1622. 39059996

[27] Fisher R Alderwick H . New plan for supporting general practice in England. BMJ 2021;375:n2585. 10.1136/bmj.n2585. 34686537

[28] Salisbury H . Helen Salisbury: The inverse care law in the digital age. BMJ 2019;364:l308. 10.1136/bmj.l308. 30700404

[29] NHS England. Appointments in General Practice. https://digital.nhs.uk/data-and-information/publications/statistical/appointments-in-general-practice.

[30] ONS. Measuring NHS experience and satisfaction across the UK, 2024 https://www.ons.gov.uk/peoplepopulationandcommunity/healthandsocialcare/healthcaresystem/articles/measuringnhsexperienceandsatisfactionacrosstheuk/2024-05-30

[31] NHS Digital. General Practice Workforce: Official statistics. https://digital.nhs.uk/data-and-information/publications/statistical/general-and-personal-medical-services. Accessed 2025.

[32] Turas Data Intelligence. Intelligence on the health care workforce in Scotland. https://turasdata.nes.nhs.scot/. Accessed 2025.

[33] Scottish Government. GMS contract: 2018. https://www.gov.scot/publications/gms-contract-scotland/pages/9/.

[34] Blumenthal D Gumas E Shah A . Mirror, mirror 2024: a portrait of the failing US health system. The Commonwealth Fund, 2024.

[35] OECD . Realising the Potential of Primary Health Care. OECD Publishing, 2020.

[36] Atherton H Leach H Mortell R Parsons J . What patients want from access to UK general practice: systematic review. Br J Gen Pract 2025;75:e526-32. 10.3399/BJGP.2024.0582. 40107729 PMC12729050

[37] Goff M Hindi A Hammond J Jacobs S . Access or continuity: a zero sum game? A systematic review of the literature examining the relationship between access and continuity in primary healthcare. BMC Prim Care 2025;26:202. 10.1186/s12875-025-02860-8. 40604407 PMC12217832

[38] Palmer W, Hemmings N, Rosen R, et al. Improving access and continuity in general practice: Practical and policy lessons. Online: Nuffield Trust, 2018.

[39] House of Commons Health and Social Care Committee. The future of general practice: Fourth Report of Session 2022-23, 2022.

[40] Sinnott C Ansari A Price E . Understanding access to general practice through the lens of candidacy: a critical review of the literature. Br J Gen Pract 2024;74:e683-94. 10.3399/BJGP.2024.0033 38936884 PMC11441605

[41] Department of Health and Social Care. Managing healthcare easy as online banking with revamped NHS App - Press Release, 2025. https://www.gov.uk/government/news/managing-healthcare-easy-as-online-banking-with-revamped-nhs-app.

[42] Kraus S Jones P Kailer N . Digital Transformation: An Overview of the Current State of the Art of Research. SAGE Open 2021;11:21582440211047576. 10.1177/21582440211047576.

[43] Dakin FH Rybczynska-Bunt S Rosen R Clarke A Greenhalgh T . Access and triage in contemporary general practice: A novel theory of digital candidacy. Soc Sci Med 2024;349:116885. 10.1016/j.socscimed.2024.116885. 38640742

[44] Nussbaum C Massou E Fisher R Morciano M Harmer R Ford J . Inequalities in the distribution of the general practice workforce in England: a practice-level longitudinal analysis. BJGP Open 2021;5:BJGPO.2021.0066. 10.3399/BJGPO.2021.0066. 34404634 PMC8596307

[45] Husain L Finlay T Husain A Wherton J Hughes G Greenhalgh T . Developing user personas to capture intersecting dimensions of disadvantage in older patients who are marginalised: a qualitative study. Br J Gen Pract 2024;74:e250-7. 10.3399/BJGP.2023.0412. 38242714 PMC10947364

[46] Lynch H Lunn AD Blythe J . What works: Mitigating inequalities in telephone and digital triage for primary health care. Health Equity Evidence Centre, 2024.

[47] Newbould J Hocking L Sidhu M Daniel K . Digital First Primary Care for those with multiple long-term conditions: a rapid review of the views of stakeholders. Health Soc Care Deliv Res 2024;12:1-68. 10.3310/AWBT4827. 39056123

[48] The Health Foundation. General Practice Data Dashboard. https://www.health.org.uk/reports-and-analysis/analysis/general-practice-data-dashboard, 2025.

[49] Pettigrew LM Bharmal AV Akl S . Trends in the shortfall of English NHS general practice doctors: repeat cross sectional study. BMJ 2025;390:e083978. 10.1136/bmj-2024-083978. 40962408 PMC12442227

[50] Skoogh A Thürer M Subramaniyan M . Throughput bottleneck detection in manufacturing: a systematic review of the literature on methods and operationalization modes. Prod Manuf Res 2023;11:2283031. 10.1080/21693277.2023.2283031.

[51] Beech J Fraser C Gardner T . Stressed and overworked: What the Commonwealth Fund’s 2022 International Health Policy Survey of Primary Care Physicians in 10 Countries Means for the UK. Health Foundation, 2023.

[52] Walker B, Sutton M, Bullen H, et al. Final Report on the 12th National GP Worklife Survey, 2025.

[53] Chen J Kasteridis P Anteneh Z . Less continuity with more complaints: a repeated cross-sectional study of the association between relational continuity of care and patient complaints in English general practice. BMJ Qual Saf 2025;bmjqs-2025-018989. 10.1136/bmjqs-2025-018989. 41057254 PMC13217021

[54] Engström SG André M Arvidsson E Östgren CJ Troein M Borgquist L . Personal GP continuity improves healthcare outcomes in primary care populations: a systematic review. Br J Gen Pract 2025;75:e518-25. 10.3399/BJGP.2024.0568. 40355250 PMC12729051

[55] Hull SA Williams C Schofield P Boomla K Ashworth M . Measuring continuity of care in general practice: a comparison of two methods using routinely collected data. Br J Gen Pract 2022;72:e773-9. 10.3399/BJGP.2022.0043. 35995578 PMC9423043

[56] Barker I Steventon A Deeny SR . Association between continuity of care in general practice and hospital admissions for ambulatory care sensitive conditions: cross sectional study of routinely collected, person level data. BMJ 2017;356:j84. 10.1136/bmj.j84. 28148478

[57] Forbes LJ Forbes H Sutton M Changes in patient experience associated with growth and collaboration in general practice: observational study using data from the UK GP Patient Survey. *Br J Gen Pract* 2020;bjgp20X713429 10.3399/bjgp20X713429 PMC7643819 33139333

[58] Baxter S Johnson M Chambers D Sutton A Goyder E Booth A . The effects of integrated care: a systematic review of UK and international evidence. BMC Health Serv Res 2018;18:350. 10.1186/s12913-018-3161-3. 29747651 PMC5946491

[59] Lloyd T Beech J Wolters A . Briefing: Realising the potential of community-based Multidisciplinary teams insights from evidence. Improvement Analytics Unit NHS England and The Health Foundation, 2023 10.37829/HF-2023-IAU01.

[60] Reeves S Pelone F Harrison R Goldman J Zwarenstein M . Interprofessional collaboration to improve professional practice and healthcare outcomes. Cochrane Database Syst Rev 2017;6:CD000072. 10.1002/14651858.CD000072.pub3. 28639262 PMC6481564

[61] de Dumast L Moore P Snell KI Marshall T . Trends in clinical workload in UK primary care 2005-2019: a retrospective cohort study. Br J Gen Pract 2024;74:e659-65. 10.3399/BJGP.2023.0527 38621809 PMC11388090

[62] Kajaria-Montag H Freeman M Scholtes S . Continuity of care increases physician productivity in primary care. Manage Sci 2024;70:7943-60 10.1287/mnsc.2021.02015.

[63] Poreschack LM Kajaria-Montag H Scholtes S . Strength in Teams: Named Physician Pairs Improve Continuity of Care in Primary Care Amid Workforce Pressures. *Available at* SSRN 5022396 2024 10.2139/ssrn.5022396.

[64] Marshall T Scholtes S Wyatt S . What makes general practice work: the role of continuity in efficient and sustainable primary care. Br J Gen Pract 2025;75:373-6. 10.3399/BJGP.2025.0038 40744745 PMC12729030

[65] Tallack C Charlesworth A Kelly E . The bigger picture: learning from two decades of changing NHS care in England. Health Foundation, 2020 10.37829/HF-2020-RC10.

[66] Monitor. Walk-in centre review: final report and recommendations: Monitor London, 2014.

[67] Sinnott C Moxey JM Marjanovic S . Identifying how GPs spend their time and the obstacles they face: a mixed-methods study. Br J Gen Pract 2022;72:e148-60. 10.3399/BJGP.2021.0357. 34844920 PMC8813099

[68] Frangeskou M Lewis MA Vasilakis C . Implementing standardised flow: navigating operational and professional dependencies. Int J Oper Prod Manage 2020;40:1177-99. 10.1108/IJOPM-06-2019-0493.

[69] Yang M Bishop A Sussex J Roland M Jowett S Wilson ECF . Economic evaluation of patient direct access to NHS physiotherapy services. Physiotherapy 2021;111:40-7. 10.1016/j.physio.2020.12.005. 33785196

[70] Martin SA Johansson M Heath I Lehman R Korownyk C . Sacrificing patient care for prevention: distortion of the role of general practice. BMJ 2025;388:e080811. 10.1136/bmj-2024-080811. 39837625

[71] NHS England. Social Prescribing. https://www.england.nhs.uk/personalisedcare/social-prescribing/ accessed 5 August 2025.

[72] Hiam L Klaber B Sowemimo A Marmot M . NHS and the whole of society must act on social determinants of health for a healthier future. BMJ 2024;385:e079389. 10.1136/bmj-2024-079389. 38604669

